# Alanine Aminotransferase Is Associated with an Adverse Nocturnal Blood Glucose Profile in Individuals with Normal Glucose Regulation

**DOI:** 10.1371/journal.pone.0056072

**Published:** 2013-02-12

**Authors:** Jian Zhou, Yifei Mo, Hong Li, Xingwu Ran, Wenying Yang, Qiang Li, Yongde Peng, Yanbing Li, Xin Gao, Xiaojun Luan, Weiqing Wang, Weiping Jia

**Affiliations:** 1 Department of Endocrinology and Metabolism, Shanghai Jiao Tong University Affiliated Sixth People’s Hospital, Shanghai Diabetes Institute, Shanghai Clinical Center for Diabetes, Shanghai, China; 2 Department of Endocrinology and Metabolism, Sir Run Run Shaw Hospital, College of Medicine, Zhejiang University, Hangzhou, China; 3 Department of Endocrinology and Metabolism, West China Hospital, Sichuan University, Chengdu, China; 4 Department of Endocrinology and Metabolism, China-Japan Friendship Hospital, Beijing, China; 5 Department of Endocrinology and Metabolism, The Second Affiliated Hospital of Harbin Medical University, Harbin, China; 6 Department of Endocrinology and Metabolism, Shanghai Jiao Tong University Affiliated First People’s Hospital, Shanghai, China; 7 Department of Endocrinology and Metabolism, The First Affiliated Hospital of Sun Yat-Sen University, Guangzhou, China; 8 Department of Endocrinology and Metabolism, Fudan University Affiliated Zhongshan Hospital, Shanghai, China; 9 Department of Endocrinology and Metabolism, The First People’s Hospital of Foshan, Foshan, China; 10 Shanghai Clinical Center for Endocrine and Metabolic Diseases, Shanghai Institute of Endocrinology and Metabolism, Ruijin Hospital, Shanghai Jiao Tong University School of Medicine, Shanghai, China; University of Hong Kong, China

## Abstract

**Objective:**

Although the association between alanine aminotransferase (ALT) levels and risk of type 2 diabetes is well-studied, the effects of slightly increased ALT levels within the normal range on the temporal normal glucose profile remains poorly understood.

**Methods:**

A total of 322 Chinese subjects without impaired glucose tolerance or previous diagnoses of diabetes were recruited for study from 10 hospitals in urban areas across China. All subjects wore a continuous glucose monitoring (CGM) system for three consecutive days. The diurnal (06∶00–20∶00) and nocturnal (20∶00–06∶00) mean blood glucose (MBG) levels were calculated. Subjects were stratified by ALT quartile level and correlation analyses were performed.

**Results:**

The median ALT level was 17 IU/L, and subjects with ALT ≥17 IU/L had higher nocturnal MBG level than those with ALT <17 IU/L (*P*<0.05). Nocturnal MBG was positively correlated with ALT levels (Pearson correlation analysis: r = 0.187, *P* = 0.001), and the correlation remained significant after correction for the homeostatic model assessment of insulin resistance index (HOMA-IR) (r = 0.105, *P* = 0.041). No correlations were found between diurnal MBG and ALT, and nocturnal or diurnal MBG and aspartate aminotransferase or gamma-glutamyltransferase (all, *P*>0.05). Multivariate stepwise regression analysis of elevated nocturnal MBG identified increased HOMA-IR, elevated ALT levels, and decreased homeostatic model assessment of ß-cell function as independent factors (all, *P*<0.05).

**Conclusions:**

Mildly elevated ALT levels, within the normal range, are associated with unfavorable nocturnal glucose profiles in Chinese subjects with normal glucose regulation.

## Introduction

The liver plays an important role in maintaining normal glucose concentrations during fasting and postprandial periods. The processes of glycogenesis, glycogenolysis, glyconeogenesis, lipid metabolism, and insulin degradation all take place within the liver, any of which may be detrimentally impacted by pathogenic, toxic or metabolic insults to the organ. Accumulation of fat in the liver in the absence of substantial alcohol intake, a condition known as nonalcoholic fatty liver disease (NAFLD), manifests as a broad spectrum of liver disorders, ranging from simple steatosis and steatohepatitis to advanced fibrosis and cirrhosis [1]. Moreover, epidemiological studies have implicated NAFLD in obesity, insulin resistance [2], type 2 diabetes [3], metabolic syndrome [4], [5], and cardiovascular diseases [6], [7].

Quantitative analysis of liver enzymes is a sensitive method to detect the presence of liver fat deposition in the absence of clinical symptoms and signs of liver disease; such an approach has emerged as a routinely used indicator of NAFLD [8], [9]. Also, alanine aminotransferase (ALT), aspartate aminotransferase (AST), and gamma-glutamyltransferase (GGT) have been characterized as predictors of type 2 diabetes [10]–[12]. A prospective study in Pima Indians showed that a higher ALT concentration is associated with a decline in hepatic insulin sensitivity, thereby promoting the development of type 2 diabetes [11]. High levels of ALT are not only frequently observed in association with raised insulin levels [13], but also with the development of metabolic syndrome [14] and cardiovascular diseases [15].

Although the association of elevated ALT with higher risk for type 2 diabetes has been frequently reported, a clear association between ALT levels, especially within its normal range, and the glucose profile remains poorly understood. In this study, we measured ALT levels in a large multi-center cohort of individuals with normal glucose regulation (NGR) to determine the association between ALT elevations and unfavorable glucose patterns. In addition, we evaluated the nocturnal and diurnal glucose profiles to investigate whether the extent of a slight elevation in ALT levels affected either profile more substantially. The associations observed between enhanced ALT levels, within the normal range, and unfavorable glucose patterns may provide insights into the underlying mechanisms.

## Methods

### Ethics Statement

The original study protocols and use of collected data for subsequent analyses received approval from the Ethics Committees of the following hospitals: Shanghai Jiao Tong University Affiliated Sixth People’s Hospital, Sir Run Run Shaw Hospital, West China Hospital, China-Japan Friendship Hospital, The Second Affiliated Hospital of Harbin Medical University, Shanghai Jiao Tong University Affiliated First People’s Hospital, The First Affiliated Hospital of Sun Yat-Sen University, Fudan University Affiliated Zhongshan Hospital, The First People’s Hospital of Foshan and Ruijin Hospital Shanghai Jiao Tong University School of Medicine, in accordance with the principle of the Helsinki Declaration II. All the above Ethnic Committees approved this study. Written informed consent was obtained from each participant.

### Study Population

This study is a subgroup analysis of a dataset from an earlier study [16]. A total of 588 subjects without related metabolic disorders were screened from 10 academic hospitals in China between October 2007 and July 2008 based on the following inclusion criteria: (1) clinically stable condition with no previous medical history of diabetes, hypertension, dyslipidemia, coronary artery diseases, or cerebral stroke; (2) fasting plasma glucose (FPG) of <5.6 mmol/L and 2 h plasma glucose (2 h PG) of <7.8 mmol/L in response to a 75 g oral glucose tolerance test (OGTT) (according to the 2007 American Diabetes Association (ADA) diabetes diagnostic criteria [17]); (3) normal body mass index (BMI, 18.5–24.9 kg/m2) [18], (4) triglycerides (TG) of <1.7 mmol/L and high-density lipoprotein cholesterol (HDL-C) of ≥1.04 mmol/L (according to the 2007 Chinese guidelines on prevention and treatment of dyslipidemia [19]); and (5) systolic blood pressure of <140 mmHg and diastolic blood pressure of <90 mmHg. Individuals were denied enrollment according to: (1) use of any medication that may affect liver function or glucose metabolism within one month prior to the study; (2) enhanced (above the hospital reference intervals) levels of ALT (>69 IU/L), AST (>46 IU/L), or GGT(>58 IU/L); (3) positivity for either hepatitis B virus surface antigen or hepatitis C virus antibody, or a history of viral hepatitis, autoimmune chronic hepatitis, drug-induced liver disease, or any other congenital liver disease; or (4) daily alcohol consumption >20 g/day (men) or 10 g/day (women), or history of alcohol abuse. Study enrollees were required to report use of any medications during the study period; if medications that adversely affect liver function were reported, the participant’s data was excluded from all study analyses. Among 588 subjects, 445 subjects were enrolled for continuous glucose monitoring (CGM). Eleven subjects were excluded for final analysis due to the CGM system signal interruption or not meeting the accuracy requirements. Of the remaining 434 subjects, data from 322 subjects were incorporated into the statistical analysis as designed for the present subgroup analysis. Subjects were divided into four subgroups according to the quartile distribution of ALT values as follows: <13 IU/L; 13–16 IU/L; 17–22 IU/L; >22 IU/L.

### Continuous Glucose Monitoring (CGM)

Subcutaneous interstitial glucose was monitored continuously for three consecutive days using a CGM system (Medtronic Inc., Northridge, CA, USA). The sensor of the CGM system was inserted on day 0 and removed on day 3, and provided a total of 288 blood glucose values from 5 min intervals over 24 h. Over the three-day period, the CGM system was calibrated daily by entering a minimum of four capillary blood glucose readings that were obtained with a SureStep blood glucose meter (LifeScan, Milpitas, CA, USA). Diurnal and nocturnal phases were defined as 06∶00–20∶00 and 20∶00–06∶00, respectively. The 24 h mean blood glucose (MBG) level was calculated from the 288 consecutive sensor readings over a 24 h period. The 24 h MBG, diurnal MBG and nocturnal MBG were based on the mean values taken on days 1 and 2.

The subjects were instructed to adhere to a standard diet during the three-day period of CGM sensor monitoring. The diet was designed to ensure a total daily caloric intake of 30 kcal/kg/day, with 50% of calories coming from carbohydrates, 15% from proteins, and 35% from fats. Written instructions were provided to achieve the appropriate caloric content and to guide the consumption times, which included breakfast (20% of daily calories, 06∶30–07∶30), lunch (40%, 11∶30–12∶30), and dinner (40%, 18∶00–19∶00).

### Laboratory Examinations

On a separate day from three-day CGM measurement, fasting venous blood sample was drawn on 06∶00 after a 10 hour overnight fast to test the laboratory examinations; and then each participant had a 75 g OGTT test. Plasma glucose was measured using the glucose oxidase method. Hepatic biomarkers, including ALT, AST, GGT and alkaline phosphatase (AKP); renal function biomarkers including blood urea nitrogen (BUN), plasma creatinine, and uric acid; TG, total cholesterol (TC), HDL-C, and low-density lipoprotein cholesterol (LDL-C) were determined by standard enzymatic methods on a 7600–020 automatic biochemistry analyzer (Hitachi High-Technologies, Tokyo, Japan). Serum insulin was measured by radioimmunoassay (Linco Research, St. Charles, MO, USA; intra- and inter-assay CVs <10%). The homeostatic model assessment of ß-cell function (HOMA-ß) was calculated as baseline insulin secretion parameter [20], whereas the early insulinogenic index, calculated as the ratio of incremental insulin to glucose responses over the first 30 min of OGTT (ΔI_30_/ΔG_30_ = (30 min INS-FINS)/(30 min PG-FPG)), was applied to evaluate early-phase insulin secretion [21]. Insulin sensitivity was calculated by homeostatic model assessment of insulin resistance index (HOMA-IR) [20] and Cederholm insulin sensitivity index (ISIc) [22].

### Statistical Analysis

All statistical analyses were carried out with the SPSS software suite, version 13.0 (SPSS Inc., Chicago, IL, USA). Normally distributed data are presented as mean ± SD, and skewed variables are presented as median (interquartile range: 25^th^ to 75^th^ percentile). Clinical characteristics with normal distribution were compared among four groups using one-way analysis of variance, while those with non-normal distribution were compared by the Kruskal-Wallis test. Pearson correlation coefficients were calculated to assess the strength of correlations between each of the nocturnal MBG levels and the various parameters. To identify independent factors that influence nocturnal and diurnal MBG, multiple stepwise regression analysis with adjustments for potential confounding factors was used. Statistical significance was indicated by a two-tailed *P*-value <0.05.

## Results

### Subject Characteristics According to ALT Category

The 322 study participants included 159 healthy men and 163 healthy women, with total group mean age of 43±14 years (range: 20–69) and BMI of 22.18±2.16 kg/m^2^. The total group median ALT level was 17 IU/L. The four ALT subgroups were composed of 80 subjects with <13 U/L, 81 subjects with 13–16 U/L, 80 subjects with 17–22 U/L, and 81 subjects with >22 U/L; As shown in Table 1, age, BMI, waist circumference, systolic blood pressure, diastolic blood pressure, TC, TG, LDL-C, AST, AKP, GGT, uric acid, FPG, OGTT 30 min plasma glucose, HOMA-IR increased and ISIc levels decreased as ALT levels increased for all four groups (all, *P*<0.05).

**Table 1:** Characteristics of study subjects stratified by ALT quartile.

		ALT quartile: IU/L				
	Total cohort	Q1: <13	Q2∶13∼16	Q3∶17∼22	Q4: >22	*P*-value
Subjects, n	322	80	81	80	81	-
Age, years	43±14	36±13	44±15	46±14	44±14	0.001
BMI, kg/m^2^	22.18±2.16	21.57±1.86	21.64±1.96	22.34±1.96	23.18±2.45	<0.001
Waist circumference, cm	78.60±7.95	77.14±7.18	76.75±7.70	78.36±7.98	82.00±7.90	<0.001
SBP, mmHg	113±13	110±13	111±14	115±11	116±11	0.01
DBP, mmHg	73±8	72±8	71±8	75±7	75±7	0.001
TC, mmol/L	4.64±0.87	4.41±0.80	4.58±0.91	4.79±0.92	4.76±0.80	0.023
TG, mmol/L	1.04±0.38	0.89±0.32	1.02±0.37	1.10±0.35	1.12±0.38	<0.001
HDL-C, mmol/L	1.51±0.36	1.54±0.36	1.54±0.35	1.51±0.36	1.46±0.37	0.441
LDL-C, mmol/L	2.77±0.86	2.56±0.81	2.68±0.87	2.88±0.90	2.94±0.78	0.015
AST, IU/L	22.65±0.36	17.86±5.14	20.81±5.18	22.98±7.98	28.74±9.48	<0.001
AKP, IU/L	63.50±18.37	55.58±17.46	63.26±19.20	63.93±17.02	71.00±16.52	<0.001
GGT, IU/L	17.69±10.07	12.16±6.30	14.19±5.72	18.76±9.62	25.54±12.73	<0.001
Uric acid	290.23±70.13	261.79±63.01	286.66±59.90	283.85±69.27	326.98±72.93	<0.001
FPG, mmol/L	4.86±0.40	4.71±0.42	4.84±0.44	4.91±0.40	4.94±0.43	<0.001
30 min PG, mmol/L	8.04±1.48	7.64±1.27	7.80±1.30	8.32±1.44	8.41±1.74	0.01
2 h PG, mmol/L	5.48±1.13	5.30±0.90	5.43±1.11	5.68±1.26	5.52±1.20	0.235
HOMA-IR	2.23 (1.71–2.93)	1.93 (1.52–2.50)	2.21 (1.71–2.68)	2.22 (1.67–3.06)	2.73 (1.96–3.24)	<0.001
HOMA-ß	149.84(113.23–212.21)	165.21(115.58–231.00)	146.72(109.68–195.07)	140.18(107.47–202.93)	164.29(119.02–225.12)	0.227
ISIc	86.90 (72.97–99.13)	94.56 (82.66–104.83)	86.60 (74.15–97.30)	85.28 (67.39–97.89)	78.48 (64.93–96.01)	0.044
ΔI_30_/ΔG_30_	16.99 (10.62–27.26)	16.49 (10.61–22.00)	18.29 (10.81–29.49)	15.02 (9.58–26.83)	18.68 (11.01–32.13)	0.153

Data are means ± SD or median (interquartile range). BMI, body mass index; SBP, systolic blood pressure; DBP, diastolic blood pressure; TC, total cholesterol; TG, triglyceride; HDL-C, high-density lipoprotein cholesterol; LDL-C, low-density lipoprotein cholesterol; AST, aspartate transaminase; AKP, alkaline phosphatase; GGT, gamma-glutamyltransferase; FPG, fasting plasma glucose; 30 min PG, OGTT 30-min plasma glucose; 2 h PG, OGTT 2-h plasma glucose; HOMA-IR, homeostasis model assessment-insulin resistance; HOMA-ß, homeostasis model assessment-pancreatic ß-cell function; ISIc, insulin sensitivity index resulting from Cederholm formula; ΔI_30_/ΔG_30_ = (30 min INS-FINS)/(30 min PG-FPG).

### Nocturnal and Diurnal CGM Profiles of ALT-defined Subgroups

**Figure 1:**
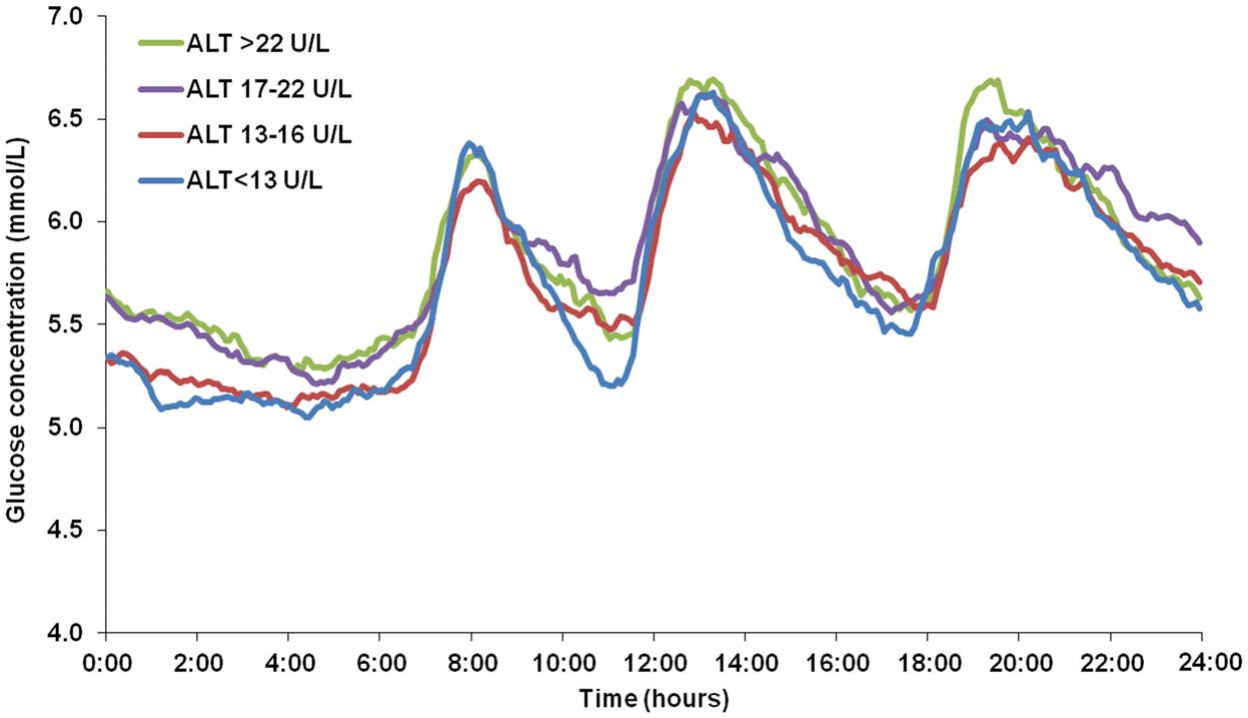
Continuous glucose profiles representing mean data from 24 hours of monitoring. The figure shows that among four subgroups according to the quartile distribution of ALT values (<13 IU/L, 13–16 IU/L, 17–22 IU/L, >22 IU/L), subjects with ALT between 17–22 IU/L and ALT >22 IU/L had higher nocturnal MBG level than the other two groups (*P*<0.05).

**Figure 2:**
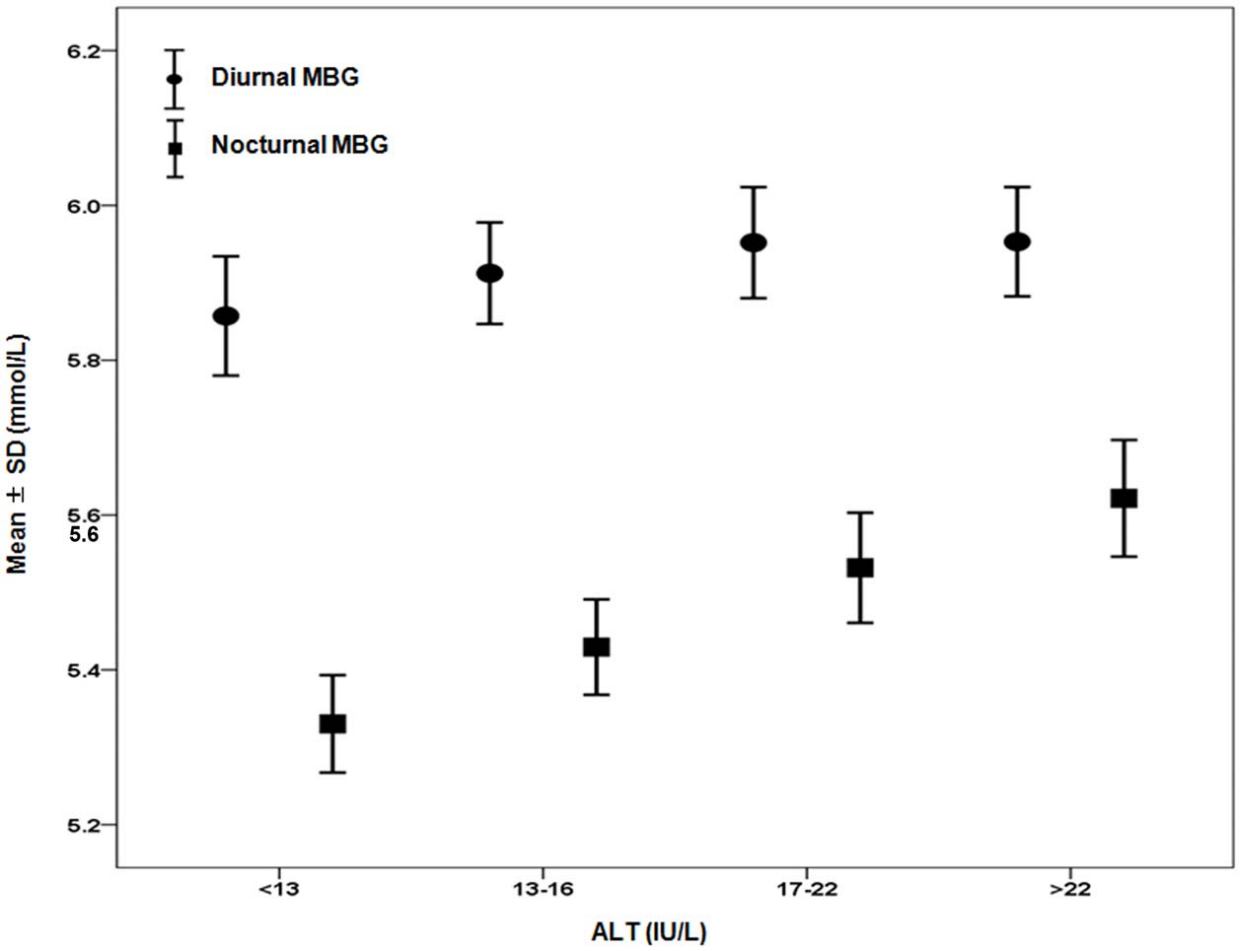
Comparison of nocturnal or diurnal MBG levels among four quartile-specific ALT subgroups. Subjects were divided to four subgroups according to the quartile distribution of ALT values (<13 IU/L, 13–16 IU/L, 17–22 IU/L, >22 IU/L). When the nocturnal MBG of four subgroups were compared after correcting for age and gender, subjects with ALT between 17–22 IU/L and ALT >22 IU/L presented significant higher MBG than the other two groups (both, *P*<0.05). Black circles = diurnal MBG; black squares = nocturnal MBG. Definition: diurnal (06∶00–20∶00); nocturnal (20∶00–06∶00) Abbreviation: MBG, mean blood glucose.

The 24 h glucose fluctuation pattern was generally similar among the four ALT-defined subgroups. The overall pattern featured three postprandial peaks and a single nocturnal nadir; however, the mean blood glucose value during the night was significantly higher for subjects in the ALT ≥17 IU/L subgroups (*P*<0.05) (Figure 1). After age- and sex-adjustment, the nocturnal MBGs of the ALT 17–22 IU/L and >22 IU/L subgroups were confirmed to be significantly higher than the MBGs of the other two groups (both, *P*<0.05). The values of nocturnal blood glucose in four subgroups were as follows: 5.33±0.55 mmol/L, 5.43±0.57 mmol/L, 5.53±0.64 mmol/L and 5.62±0.68 mmol/L. The values of diurnal blood glucose in four subgroups were: 5.86±0.67 mmol/L, 5.91±0.60 mmol/L, 5.95±0.64 mmol/L and 5.95±0.64 mmol/L (data in mean ± SD). No significant difference was found among the four ALT subgroups for the diurnal blood glucose (all, *P*>0.05) (Figure 2).

### Factors Influencing Blood Glucose Profiles in NGR

Pearson correlation analysis indicated that nocturnal MBG was positively correlated with ALT levels (r = 0.187, *P* = 0.001), and the correlation remained significant after correction for HOMA-IR (r = 0.105, *P* = 0.041). In addition, the Pearson correlation analysis confirmed the lack of correlation between diurnal MBG and ALT, and nocturnal or diurnal MBG and AST or GGT (all, *P*>0.05).

To determine the independent parameters that correlated with nocturnal MBG, the variables that significantly correlated with nocturnal MBG in the univariate analysis were tested in a multiple stepwise regression analysis (Table 2). In model 1, the independent variables were age, sex, BMI, waist circumference, blood pressure, TC, TG, HDL-C, LDL-C, ALT, AST, AKP, GGT, BUN, plasma creatinine, and uric acid. The multivariate regression analysis identified elevated ALT levels, elevated LDL-C levels, and older age as predictive factors of increased nocturnal MBG. In model 2, HOMA-β, ΔI_30_/ΔG_30_, HOMA-IR, and ISIc were added as the independent variables. The multivariate regression analysis identified elevated HOMA-IR, decreased HOMA-β and elevated ALT levels as predictors of elevated nocturnal MBG. Analysis of diurnal MBG with the above-described model 2 identified age (standardized ß = 0.211, *P*<0.001) and HOMA-IR (standardized ß = 0.263, *P*<0.001) as positive predictive factors, and HOMA-β (standardized ß = –0.134, *P* = 0.025) and ΔI_30_/ΔG_30_ (standardized ß = – 0.152, *P* = 0.009) as negative predictive factors.

**Table 2:** Independent effectors of nocturnal mean blood glucose.

	Standardized ß	*t*	*P-* value
**Model 1**			
ALT	0.169	3.010	0.002
LDL-C	0.316	3.452	0.001
Age	0.121	2.034	0.033
**Model 2**			
HOMA-IR	0.312	5.122	<0.001
HOMA-β	−0.189	−3.241	0.001
ALT	0.112	1.961	0.041

For Model 1, independent variables were age, gender, body mass index, waist circumference, blood pressure, triglycerides, total cholesterol, high-density lipoprotein cholesterol, low-density lipoprotein cholesterol (LDL-C), alanine aminotransferase (ALT), blood urea nitrogen, plasma creatinine and uric acid. For Model 2, homeostatic model assessment of insulin resistance index (HOMA-IR), homeostatic model assessment of ß-cell function (HOMA- ß), ΔI30/ΔG30 and Cederholm insulin sensitivity index were included in addition to the previously described independent variables in Model 1.

### Factors Influencing ALT Levels in NGR

Multivariate regression analysis revealed that increased waist circumference, uric acid and HOMA-IR and decreased HOMA-β were predictors of elevated ALT levels (all, *P*<0.05) among potential confounding risk factors as age, gender, BMI, waist circumference, blood pressure, TC, TG, HDL-C, LDL-C, BUN, plasma creatinine, uric acid, HOMA-β, ΔI_30_/ΔG_30_, HOMA-IR, and ISIc (Table 3).

**Table 3:** Independent effectors of ALT levels.

	Standardized ß	*t*	*P*-value
Waist circumference	0.172	2.845	0.005
Uric acid	0.248	4.260	<0.001
HOMA-IR	0.264	4.507	<0.001
HOMA-β	−0.126	−2.226	0.027

Independent variables were age, gender, body mass index, waist circumference, blood pressure, triglycerides, total cholesterol, high-density lipoprotein cholesterol, low-density lipoprotein cholesterol, blood urea nitrogen, plasma creatinine and uric acid, homeostatic model assessment of insulin resistance index (HOMA-IR), homeostatic model assessment of ß-cell function (HOMA-ß), ΔI_30_/ΔG_30_ and Cederholm insulin sensitivity index.

## Discussion

The present study demonstrated that slightly elevated ALT concentration within its normal range is an independent risk factor for an adverse nocturnal glucose profile in Chinese subjects without previously diagnosed diabetes or impaired glucose tolerance. In addition, although the association between ALT and nocturnal MBG was attenuated by adjusting for HOMA-IR, the correlation remained significant, which implicates hepatic insulin resistance as part of the potential underlying contributor to this biochemical process.

Previous cross-sectional studies have demonstrated associations between fatty liver and metabolic abnormalities, including insulin resistance [2], [23] and diabetes [24], [25]. The most common clinical methods used to diagnose and estimate liver fat deposition include detection of liver enzymes, ultrasonography, and ^1^H-magnetic resonance spectroscopy (^1^H-MRS). Among these methods, the liver marker assays are the most inexpensive, technically simple, and convenient (the test sample – blood – is often routinely collected in clinical settings). Meanwhile, elevated ALT levels are considered the more accurate indicator of increases in liver fat content [8], as compared to AST and GGT which are also found in other tissues and therefore less specific markers of NAFLD. Elevated ALT levels are present in and correlated to a number of other diseases, including late-onset diabetes [11], [26], [27], insulin resistance [11], metabolic syndrome [28], and atherosclerosis [29]. For example, the long-term (20 year follow-up) community-based study revealed an association between elevated ALT levels and subsequent development of metabolic syndrome and diabetes mellitus [30]. The results from the study described herein, which used ALT as a marker of NAFLD in NGR patients, indicated that elevated ALT levels can significantly influence temporal glucose fluctuations, specifically those in the nocturnal phase. This finding agrees with our previous research, which showed that as liver fat content increases, the adverse nocturnal glucose profiles appear earlier than the adverse diurnal glucose profiles [31].

In the current study, NGR subjects were stratified according to the quartile distribution of ALT. Subjects with ALT ≥17 IU/L had higher nocturnal MBG than subjects with ALT <17 IU/L, suggesting that slight elevations in ALT, even within the normal range, could influence an unfavorable blood glucose profile. It is possible that the currently used normal range for liver enzymes does not distinguish individuals with NAFLD. Prati et al. reported that the ALT <19 IU/L is appropriate for women and <30 IU/L is appropriate for men [32]. Suh et al. reported a similar sex-distinctive range for Korean adults to indicate metabolic syndrome, with high-normal ALT levels being ≥27 IU/L in men and ≥15 IU/L in women [33]. An ethnic-distinctive range may be appropriate as well, as Gao et al. demonstrated that the optimal cut-off values for ALT to identify metabolic syndrome in Chinese patients was 26 IU/L in men and 20 IU/L in women [34]. Since the previous literature has suggested that ALT reference range should differ for men and women, we performed statistical analysis for all the data with sex adjustment in the present study and found that gender has no impact on the association between plasma ALT levels and nocturnal MBG in observed subjects.

The mechanisms underlying the correlation between elevated ALT levels and unfavorable glucose profile are not completely understood. Based on the results from the current study, hepatic insulin resistance appears to contribute to this process, but the precise factors and causative/responsive molecular networks remain unknown. We may gain insight into this biological interplay by considering the known mechanisms of elevated liver transaminase levels and insulin resistance in the context of NAFLD [35], [36]. Liver is a major site of insulin clearance, and loss of insulin-mediated suppression of hepatic glucose production and glycogenolysis leads to an increase in hepatic glucose production [37], [38]. This process directly affects nocturnal MBG and fasting glucose levels, which may explain our finding of elevated ALT levels specifically affecting nocturnal MBG and not diurnal MBG.

The different results calculated by two multiple stepwise regression models are interesting. If hepatic insulin resistance acts as the only mediator of the association between ALT and unfavorable nocturnal glucose profile, there should be no association between ALT and nocturnal MBG after controlling for HOMA-IR, which mainly reflects hepatic insulin resistance. However, in the present study, this association between ALT and nocturnal MBG remained after controlling for HOMA-IR, although attenuated, suggesting hepatic insulin resistance may be part of, but not the only potential underlying contributor to this biochemical process. In addition, previous literature reported that a low-grade inflammatory state can induce development of subsequent type 2 diabetes [39] and liver biopsy specimens of individuals with fatty infiltration have increased expression of inflammation-related genes [40]. Thus, it is possible that immune-related factors, especially those involved in the inflammatory response, may contribute to ALT-related fluctuations of blood glucose. Future studies should address the underlying molecular mechanisms of this process.

The current cross-sectional study involved subjects recruited from multiple hospitals across the nation. The CGM system allowed for collection of a 24 h glucose profile for each study participant, which is more comprehensive than the data collected by means of OGTT or fasting blood glucose test. However, several limitations exist within the study design that must be considered when interpreting the findings. First, neither ultrasound imaging nor liver biopsies was used to measure hepatic fat in the study participants. Also, we did not determine the inflammatory factors in this study. Lacking the measurements of NAFLD and inflammatory factors makes it difficult to justify the normal high ALT values was caused by either fatty liver or inflammation. Second, the cross-sectional design precludes determining causality between the elevated ALT levels and unfavorable glucose profiles. Third, the measurements of insulin resistance and ß-cell dysfunction were based on an indirect method (the HOMA model). To further demonstrate the role that elevated ALT plays in abnormal glucose profiles, prospective or interventional studies using the clamp technique to collect data are needed, so are the measurements of NAFLD and inflammatory factors.

### Conclusions

In conclusion, the present study demonstrated that elevated ALT levels, even within normal range, are associated with increased nocturnal glucose levels in Chinese with no previous diagnoses of diabetes or impaired glucose tolerance. Regarding the availability and simplicity of testing liver enzymes in routine clinical practice, our findings suggest the potential of ALT concentration to indicate an adverse glucose profile.

## Supporting Information

Text S1 **List of participating investigators.**
(DOC)
